# Suicidal Behavior and Haplotypes of the Dopamine Receptor Gene (*DRD2*) and ANKK1 Gene Polymorphisms in Patients with Alcohol Dependence – Preliminary Report

**DOI:** 10.1371/journal.pone.0111798

**Published:** 2014-11-21

**Authors:** Andrzej Jasiewicz, Agnieszka Samochowiec, Jerzy Samochowiec, Iwona Małecka, Aleksandra Suchanecka, Anna Grzywacz

**Affiliations:** 1 Department of Psychiatry, Pomeranian Medical University, Szczecin, Poland; 2 Institute of Psychology, Department of Clinical Psychology, University of Szczecin, Szczecin, Poland; Nathan Kline Institute for Psychiatric Research and New York School of Medicine, United States of America

## Abstract

Suicide is a significant public health issue and a major cause of death throughout the world. According to WHO it accounts for almost 2% of deaths worldwide. The etiology of suicidal behavior is complex but the results of many studies suggest that genetic determinants are of significant importance. In our study,- we have analyzed selected SNPs polymorphisms in the *DRD2* and *ANKK1* genes in patients with alcohol dependence syndrome (169 Caucasian subjects) including a subgroup of individuals (n = 61) who have experienced at least one suicide attempt. The aim of the study was to verify if various haplotypes of selected genes, comprising Taq1A, Taq1B, and Taq1D single nucleotide polymorphisms (SNP), play any role in the development of alcohol dependence and suicidal behavior. The control group comprised 157 unrelated individuals matched for ethnicity, gender,- and age and included no individuals with mental disorders. All subjects were recruited in the North West region of Poland. The study showed that alcohol dependent subjects with a history of at least one suicidal attempt were characterized by a significantly higher frequency of the T-G-A2 haplotype when compared to individuals in whom alcohol dependence was not associated with suicidal behavior (p = 0.006). It appears that studies based on identifying correlation between SNPs is the future for research on genetic risk factors that contribute to the development of alcohol addiction and other associated disorders. To sum up, there is a necessity to perform further research to explain dependencies between the dopaminergic system, alcohol use disorders and suicidal behavior.

## Introduction

Suicide is a significant public health issue and a major cause of death throughout the world. According to WHO it accounts for almost 2% of deaths worldwide [1].

The etiology of suicidal behavior is complex and the results of twin and adoption studies suggest that this issue has also genetic determinants [2],[3]. Andrei Maruśić wrote a comprehensive article about a possible history and geography of suicide attempts. He suggested that people who live within the J-curve (on the map of Europe – the countries with a higher suicide rate) could share genes,- that may not tolerate excessive amounts alcohol, the combination of which is more likely to end in suicidal behavior [4]. It is worth noticing that one of the first who pointed that some ethnic groups who have a collection of shared genes can have an elevated susceptibility to suicide was Kondrichin [5].

Since it was revealed that the low central nervous system serotonin (*5-HT*) turnover is related to suicidal behavior [6], serotonin-related genes have been the focus of several association studies[7]. These studies confirmed that suicidality, impulsiveness, and depression share a common genetic basis and biological substrate of the *5-HT* dysfunction [8],[9],[10]. Additionally, the noradrenergic and dopaminergic systems, hypothalamic-pituitary-adrenal (HPA) axis, and brain-derived neurotrophic factor have been examined for candidate genes of suicidal behavior, but no consistent associations have been identified so far [11].

Apart from the genetic factors, most studies confirm a close relationship between the risk of suicide and alcohol use disorders [12],[13]. In the study of Inskip et al.,- the lifetime risk of suicide in individuals with alcohol dependence proved higher than in those with mood disorders [14]. Moreover, the results of the most recent studies suggest that the inherited factors for suicidal susceptibility should not be considered independently from alcohol consumption [4].

Dopamine is a neurotransmitter whose role in the development of substance abuse, including alcohol dependence, is relatively best understood. It was suggested that dopamine is involved in the transmission of subjectively assessed pleasure stimulation, e.g. during a sexual intercourse, eating, drinking, or other strongly motivated behaviors, including positively enhancing effects of alcohol. According to some animal studies, a single exposure to alcohol is reflected by a higher bioelectrical activity of the ventral tegmental neurons and increased release of dopamine in nucleus accumbens. These effects were not attenuated in the case of chronic alcohol intoxication, suggesting lack of tolerance to the rewarding effects of alcohol. In contrast, the dopaminergic activity was reduced in the course of the abstinence syndrome, and therefore it can be involved in the development of aversive signs associated with abstinence, as well as it can stimulate alcohol abuse [15],[16].

Bearing the abovementioned evidence in mind, it cannot be excluded that the decreased dopaminergic activity can also be a determinant of suicide attempts. Consequently, we hypothesized that the genes involved in dopamine metabolism can be involved in common etiopathogenesis of alcohol use disorders and suicidal behavior. The results of previous studies addressing the role of the dopaminergic system in the development of alcohol dependence suggest that the dopamine receptor *DRD2* gene is a potential candidate gene involved in such a common etiopathogenic pathway [17].

The *DRD2* gene is located on the chromosome 11q23.2 [18]. Polymorphisms Taq 1A in the *ANKK1* gene connected with dopaminergic system disorders. [19],[20]. Nevertheless the Taq1B and Taq1D variants are located in introns 1 and 2 of the *DRD2*, respectively [21]. The most studied polymorphisms of these genes include the Taq1A restriction fragment length polymorphism [22] and Taq1B restriction fragment length polymorphism [23]. The Taq1A polymorphism creates two alleles: A1 (variant) and A2 [22]. Significant associations between the Taq1A polymorphism and substance dependence have been documented in some [18],[24],[25] but not all studies [26],[27]. The Taq1B polymorphism consists of two alleles: B1 (Taq1 ‘‘absent’’) and B2 (Taq1 ‘‘present’’) allele [23],[28]. The Taq1B polymorphism was also associated with alcohol dependence [29] and other substance abuses [25],[26],[30].

In this study,- we have analyzed selected SNPs polymorphisms in the *DRD2* and *ANKK1* genes in patients with the alcohol dependence syndrome including a subgroup of individuals who have experienced at least one suicide attempt. The aim of the study was to verify if various haplotypes of the selected genes, comprising the Taq1A, Taq1B, and Taq1D single nucleotide polymorphisms (SNP) play any role in the development of alcohol dependence and suicidal behavior.

## Material and Methods

This study involved a group of 169 Caucasian subjects (33±8.5 years of age, range 19–61years), with no history of psychiatric disorders other than alcohol or nicotine dependence as classified by ICD-10. This group included a subgroup of patients (n = 61) who had a documented history of at least one suicidal attempt. Other phenotypic characteristics of analyzed alcohol dependent subjects are presented in Table 1. The control group comprised 157 unrelated individuals matched for ethnicity, gender, and age (38±15.9 years, range 18–80), and included no individuals with mental disorders excluded by means of the Primary Care Evaluation of Mental Disorders (Prime MD) questionnaire. All subjects were recruited in the North West region of Poland. Alcohol use and family history of alcoholism were assessed by means of a structured interview, based on the Polish version of Semi-Structured Assessment on Genetics in Alcoholism (SSAGA).

**Table 1:** Phenotypic characteristics of the studied alcohol dependent subjects (n = 169).

Variable	n	%
**Familial history of alcohol dependence**		
No	111	65,7
Yes	57	33,7
No data	1	0,6
**Dissocial personality**		
No	109	64,5
Yes	60	35,5
**Delirium and/or seizures**		
No	117	69,2
Yes	49	29,0
No data	3	1,8
**Early onset of drinking**		
No	47	27,8
Yes	121	71,6
No data	1	0,6
**Lesch typology**		
Proper type	36	21,3
Neurotic type	44	26,0
Psychotic type	50	29,6
Organic type	26	15,4
No data	13	7,7
**Cloninger's typology**		
Type I	39	23,1
Type II	130	76,9

The study protocol was approved by the local Ethic Committee of Pomeranian Medical University and all the participants provided their written informed consent.

The genomic DNA was isolated from whole venous blood according to standard procedures. All genotyping was performed with the fluorescence resonance energy transfer Real-Time method using the Light Cycler System 2.0. The following conditions were applied in order to analyze the polymorphism of the *DRD2* receptor gene. A polymerase chain reaction (PCR) was performed with 50 ng DNA in a total volume of 20 ml containing 2 ml reaction mixture, 0.5 mM of each primer, 0.2 mM of each hybridization probe, and 2 mM MgCl_2_. According to the manufacturer's instructions, 35 cycles of denaturation (95°C for 10 min), annealing (60°C for10 sec) and extension (72°C for 15 sec) were performed. After amplification, a melting curve was generated by holding the reaction at 40°C for 20 seconds and then heating slowly up to 85°C. The fluorescence signal was plotted against temperature to provide melting curves for each sample. The following SNPs of *DRD2* receptor gene were analyzed: Taq ID rs1800498 intron 2 (C/T), Taq IB rs1079597 intron 1 (A/G). Apart from that SNP of ANKK1: Taq IA rs1800497 Ex8 (A1/A2) was analyzed[13].

The data analysis was performed using the Statistical Package for Social Sciences (SPSS) version 9.

We calculated power in G*Power 3.1 using frequencies of genotypes, haplotypes and a number of case subjects.

The differences between controls, alcoholics, and alcoholics with at least one suicide attempt were tested using the χ^2^ test and considered significant if the type 1 error was less than 5% using SPSS. The Hardy Weinberg equilibrium was calculated using the SAS computer program for Windows. The haplotype frequencies were calculated using R with Bioconductor packages haplo.stats and genetics.

## Results

Our study did not reveal any significant relationships between occurrence of alcohol dependence and alcohol dependent subjects with suicidal behavior and the genotypic and allelic frequencies of the analyzed polymorphisms.

No significant deviations from the Hardy Weinberg equilibrium was observed.

No significant statistical differences were observed in the analyzed groups: p>0.05.

The statistical power oscillated between 0.1 and 0.6 (Table 2).

**Table 2:** Frequency of genotypes and alleles of Tag ID rs1800498 intron 2 (C/T), Tag IB rs1079597 intron 1 (A/G), and Tag IA rs1800497 Ex8 (A1/A2) SNPs of *DRD2* receptor gene and ANKK1 gene in alcohol dependent subjects and alcohol dependent subjects with suicidal behavior.

*Alcohol dependent subjects*																			
	Taq1D (rs1800498)						Taq IB (rs1079597)						Taq 1 A (rs1800497)						
	C/C	C/T	T/T	C	T	H-W	A/A	A/G	G/G	A	G	H-W	A1/A1	A1/A2	A2/A2	A1		A2	H-W
Control (N = 157)	28 (18%)	80 (51%)	49 (31%)	136 (43%)	178 (57%)	0.636	7 (4%)	36 (23%)	114 (73%)	50 (16%)	264 (84%)	0.071	8 (5%)	43 (28%)	106 (67%)	59 (19%)		255 (81%)	0.198
Case(N = 169)	31 (18%)	87 (52%)	51 (30%)	149 (44%)	189 (56%)	0.565	8(5%)	53 (31%)	108 (64%)	69 (20%)	269 (80%)	0.650	8 (5%)	58 (34%)	103 (61%)	74 (22%)		264 (78%)	0.963
*p*/power	*p*−0.978/0.056			*p*−0.843			*p*−0.219/0.655			*p*−0.747			*p*−0.400/0.441				*p*−0.326		

*Alcohol dependent subjects with suicidal behavior*															
	C/C	C/T	T/T	C	T	A/A	A/G	G/G	A	G	A1/A1	A1/A2	A2/A2	A1	A2
Control(N = 157)	28 (18%)	80 (51%)	49 (31%)	136 (43%)	178 (57%)	7 (4%)	36 (23%)	114 (73%)	50 (16%)	264 (84%)	8 (5%)	43 (27%)	106 (68%)	59 (19%)	255 (81%)
Case(N = 61)	9 (15%)	28 (46%)	24 (39%)	46 (38%)	76 (62%)	0 (0%)	20 (33%)	41 (67%)	20 (16%)	102 (84%)	0 (0%)	20 (33%)	41 (67%)	20 (16%)	102 (844%)
*p*	*p*−0.512			*p*−0.287		*p*−0.102			*p*−0.905		*p*−0.169			*p*−0.560	

The alcohol dependent subjects with a history of at least one suicidal attempt were characterized by a significantly higher frequency of T-G-A2 haplotype when compared to individuals in whom alcohol dependence was not associated with suicidal behavior (*p* = 0.006; Table 3). There was no association between alcohol dependent subjects with a history of at least one suicidal attempt and the same was observed in controls (Table 4).

**Table 3:** Frequency of various haplotypes composed of Tag ID rs1800498 intron 2 (C/T), Tag IB rs1079597 intron 1 (A/G), and Tag IA rs1800497 Ex8 (A1/A2) SNPs of *DRD2* receptor gene and ANKK1 gene in alcohol dependent subjects with a history of at least one suicidal attempt (n = 61) and in individuals in whom alcohol dependence was not associated with suicidal behavior (n = 108).

Haplotype	Haplotype Score	p-value	Haplotype frequency	No suicidal behavior	Suicidal behavior
T-A-A1	−1.427	0.153	0.020	0.033	-
T-G-A1	−1.288	0.197	0.021	0.029	0.008
C-G-A2	−1.286	0.198	0.261	0.291	0.213
C-A-A1	−1.012	0.311	0.177	0.189	0.155
T-G-A2	2.705	**0.006** *	0.509	0.446	0.614

Hap-score determines a relationship between a specific haplotype and particular phenotypic quality.

*significance maintained after the Bonferroni correction was included.

power  = 0.580.

**Table 4:** Frequency of various haplotypes composed of Tag ID rs1800498 intron 2 (C/T), Tag IB rs1079597 intron 1 (A/G), and Tag IA rs1800497 Ex8 (A1/A2) SNPs of *DRD2* receptor gene and ANKK1 gene in alcohol dependent subjects with a history of at least one suicidal attempt (n = 61) and in the controls (n = 157).

Haplotype	Haplotype Score	p-value	Haplotype frequency	Controls	Suicidal behavior
C-G-A1	−1.730	0.836	0.020	0.028	-
T-G-A1	−1.349	0.177	0.026	0.033	0.008
C-A-A2	−1.291	0.196	0.023	0.029	0.008
C-G-A2	−0.878	0.379	0.242	0.253	0.213
C-A-A1	0.843	0.399	0.130	0.120	0.155
T-G-A2	1.668	0.095	0.550	0.524	0.614

Hap-score determines a relationship between a specific haplotype and particular phenotypic quality.

**power  = 0.409.**

There was a significant relationships documented by the haplotype analysis of alcohol dependence patients with suicidal attempts, which indicated usefulness of haplotype constructions in etiopathogenetic studies.

## Discussion

A number of researches, which have been performed to date, aimed at describing connections between polymorphisms of genes in the field of serotonergic and dopaminergic systems and alcohol dependency, possibly in connection with suicidal behavior as well. Typically, they are focused on single nucleotide polymorphisms (SNPs). A lot of researches performed, showed dependencies between polymorphisms of genes within the serotonergic system and suicide attempts. E.g. Wojnar et al. have found that the G/G genotype in C1018G *5HTR1A* (rs6295) gene polymorphism may be hypothetically responsible for coexistence of suicidal tendencies and relapse of alcohol use susceptibility [31]. Paredes et al. suggested that polymorphic variants on the *5-HT2A* receptor gene (A-1438G (rs6311) and T102C (rs6313) may predispose for impulsive suicidal behavior [32]. There is also a number of articles which indicate that genes from the dopaminergic system are involved in alcohol dependency. The large-scale meta-analysis from 2013 - of over 18,000 subjects included in 61 case-control studies - confirmed association between the TaqIA polymorphism (the A1 allele showed a significant association with AD) and alcohol dependence [33]. London et al. proved that ethanol decreased anxiety and fatigue in men with the A1 allele of the *ANKK1* TaqIA polymorphism, but increased in men without this allele. They made a conclusion that alcohol-induced negative reinforcement may explain a greater risk for alcohol dependence associated with the A1 allele [34]. Munafò also supported association of the individuals possessing the A1 allele of the TaqIA *ANKK1* polymorphism with alcohol dependence [35].

Most of the researches are based usually on trials conducted in order to find associations between a -single SNP or one different gene polymorphism and alcohol dependency. What is worth mentioning is that alcohol dependency syndrome is a multigene disorder. It is very different from the *BRCA1* gene, for example, when one mutation is able to cause a breast cancer. That is why an analysis of a single SNP is insufficient. The former studies have also proved that inheritance of AD is more due to genetics than other factors such as bad habits in the families or environmental factors [36]. It is established that impact of the inherited genes - no matter what sex we describe - is on the level of 50-60% [37]–[40]. That means it is worth finding genetic causes of the AD syndrome, because in the future this knowledge could be used effectively in prevention or treatment.

What we were interested in was not only the connection between genes and AD, but we also decided to focus on suicidal behavior. There is, unfortunately, a lack of comprehensive research which takes into consideration connections between the dopaminergic system, alcohol dependence and suicide attempts.

In 2009, Suda et al. described their findings by suggesting that gene polymorphisms from the dopaminergic system might have been involved in the biological susceptibility to suicide [41]. They took into consideration polymorphisms TaqIA and -141C Ins/Del. Both- the Ins allele of -141C Ins/Del and the A2 allele of TaqIA were significantly more frequent in suicide attempters. In our survey we showed that there is a significantly higher frequency of haplotype consisting of the A2 allele of TaqIA (haplotype T-G-A2) in subjects with AD and at least one suicidal attempt in comparison with those without a suicidal history. In a German research on alcoholics the - 141C Del variant of the *DRD2* might be a risk factor in a highly burdened subgroup of alcoholics with a paternal and grandpaternal history of alcohol dependence and it might contribute to the substantially higher likelihood of suicide in alcohol dependent subjects [42].

As it was said, we concentrated on the fact that AD as a multigene disorder must be connected with a number of gene polymorphisms placed in different parts of a genome. Therefore, a search for genetic changes, which could be identified as risk factors in the development of alcohol dependence, should not be focused only on analyzing associations of individual polymorphisms in candidate genes [43]–[44]. On the basis of the current knowledge it is more reasonable to seek epistatic interactions of polymorphisms located on different chromosomes [45].

Preuss et al. also analyzed the *DRD2* gene and *ANKK1* haplotypes (141 Ins/Del, Taq1B, Taq1D, rs 1079594, TaqIA). He showed a significant association with regular drinking (Ins-C-G-C-A1) and smoking (Ins-T-G-A-A2) [46]. To everything said before, we add the context of a suicidal behavior. The outcome of our research was a significant association of the T-G-A2 haplotype in subjects with AD and at least one suicide attempt compared with alcohol dependence without a suicidal history. We did not search for any connection with nicotine addiction. The results show a probability of a stronger connection between this variant of haplotype and suicidal behavior than with AD. In addition, different genes were tested for associations with suicidal attempts. Zill has chosen 20 SNPs covering the second isoform of the tryptophan hydroxylase gene (*TPH2*) to search for any role of the gene in pathophysiology of alcohol dependence or alcohol dependence - related phenotype suicidal behavior. None has been found to play a major role. Undoubtedly, further analyses are needed [47].

A small number of subjects in the analyzed group was a limitation to our research. It was an exploration sample and requires replication on a larger group.

It appears that studies based on identifying a correlation between SNPs is the future for research on genetic risk factors that contribute to the development of alcohol addiction and other associated disorders. To sum up, there is a necessity to perform further research to explain dependencies between the dopaminergic system, alcohol use disorders and suicidal behavior.
